# Methods of Rendering Sponges Aseptic

**Published:** 1890-04

**Authors:** 


### METHODS OF RENDERING SPONGES ASEPTIC.

Peren in the *Revue Generale de Clinique et de Therapeutique*, Oct. 10, 1889, gives the following method for the preparation of aseptic sponges. They are to be pounded with a wooden mallet and afterward carefully soaked in plenty of pure water. Following this, the sponges are placed in a bath of hydrochloric acid of the strength of one per cent. in order to dissolve out thoroughly calcareous particles, and when this is accomplished, they are inserted into a jar containing a solution of potassium permanganate, in which they remain six hours. For blanching they are submitted to the action of a solution containing bisulphite of soda in the proportion of 150 grains to a quart of water. Finally, all these processes being completed, they are placed in one of the following solutions for preservation:

| | | |
|---|---|---|
| R. | Carbolic acid - - - - | 15 grains. |
| | Alcohol - - - - - | 1 1-2 drachms. |
| | Water - - - - - | 1 quart. |
| or, | | |
| R. | Thymol - - - - - | 15 grains. |
| | Alcohol - - - - - | 1 1-2 drachms. |
| | Water - - - - - | 1 quart. |

*Medical News*, November 2, 1889.—*Cincinnati Medical Journal.*

DEATH FROM THE STING OF A BEE.—A Dorsetshire farmer recently died of suffocation from the œdema following the sting of a bee which got into his throat.

